# Ocular Manifestations Associated with Hematologic Malignancies: Mechanisms, Diagnosis, and Management

**DOI:** 10.3390/medsci14020230

**Published:** 2026-04-30

**Authors:** Yehan Xiao, Yaru Zou, Mingming Yang, Jing Zhang, Kyoko Ohno-Matsui, Koju Kamoi

**Affiliations:** Department of Ophthalmology & Visual Science, Graduate School of Medical and Dental Sciences, Institute of Science Tokyo, Tokyo 113-8510, Japan; xyehan2002@gmail.com (Y.X.); alicezouyaru519@gmail.com (Y.Z.); yangmm-12@outlook.com (M.Y.); zhangj.c@foxmail.com (J.Z.); k.ohno.oph@tmd.ac.jp (K.O.-M.)

**Keywords:** hematologic malignancies, leukemia, lymphoma, ocular manifestations, leukemic retinopathy, vitreoretinal lymphoma, ocular toxicity, graft-versus-host disease, opportunistic infection

## Abstract

**Background:** Hematologic malignancies (HMs), including leukemia and lymphoma, are systemic diseases that may cause a wide range of ocular manifestations. **Methods:** We searched PubMed/MEDLINE (2015-2026) and identified articles with an emphasis on clinically relevant studies and recent developments. **Results:** Clinically, ocular involvement presents with diverse manifestations, including retinal hemorrhage, vitreoretinal lymphoma, choroidal infiltration, orbital masses, treatment-related ocular toxicities, graft-versus-host disease, and secondary infectious complications. These findings may mimic other ocular diseases and consequently lead to delayed diagnosis. In some cases, ocular manifestations may represent the initial presentation of hematologic malignancies or indicate disease recurrence. Diagnostic evaluation relies on comprehensive ophthalmic examination, imaging, and laboratory analysis. Management strategies include systemic treatment of the underlying malignancy, local ocular therapy, and targeted treatment of infectious or treatment-related complications. **Conclusions:** Ocular manifestations of hematologic malignancies have significant diagnostic and prognostic implications. Early recognition, multidisciplinary collaboration, and comprehensive ophthalmic assessment are essential for timely diagnosis and optimal management. Improved awareness of disease-related, treatment-related, and infection-related ocular manifestations may facilitate earlier intervention and contribute to better visual and systemic outcomes.

## 1. Introduction

Hematologic malignancies (HMs), including leukemia, lymphoma, multiple myeloma (MM), myelodysplastic syndrome (MDS), and myeloproliferative neoplasms (MPN), comprise a heterogeneous group of malignancies originating from the hematologic system or lymphoid tissues [1]. Leukemia is classified according to cell lineage and the degree of cellular differentiation and maturation, and is broadly divided into acute lymphoblastic leukemia (ALL), acute myeloid leukemia (AML), chronic myeloid leukemia (CML), and chronic lymphocytic leukemia (CLL) [2]. Lymphomas are generally divided into Hodgkin lymphoma (HL) and non-Hodgkin lymphoma (NHL) based on histopathological characteristics. NHL comprises B-cell, T-cell, and NK-cell neoplasms [3]. From 1990 to 2021, the global incidence of HMs increased steadily, with NHL exhibiting the most pronounced rise [4]. In 2022, the global lifetime risk of incidence for HMs was 1.67%, and the corresponding lifetime risk of mortality from these malignancies was 0.98% [5].

HMs are inherently systemic diseases and may involve multiple organs, resulting in multisystem involvement. In acute ALL, common systemic manifestations include infections, bleeding tendencies, and anemia, while leukemic infiltration may also be observed in extramedullary sites including the central nervous system, liver, spleen, and eyes [6]. In recent years, advances in the understanding of the pathogenesis and biological characteristics of HMs have led to the development of novel therapeutic strategies that have markedly improved patient outcomes and substantially prolonged survival [4]. For example, the use of tyrosine kinase inhibitors (TKI) for CML has enabled low-risk patients to achieve a 10-year overall survival rate approaching 90% [7].

With the progression of research, ocular involvement has become increasingly recognized in patients with HMs. These manifestations may include retinal hemorrhages, uveitis, secondary infections, and chloromas [8,9,10]. These manifestations may not only indicate recurrence but also represent the initial presentation [11]. At the same time, certain treatments have also been reported to cause ocular adverse effects [12]. Therefore, ophthalmic examinations in patients with hematologic malignancies may facilitate the early detection of disease recurrence and infections, thereby contributing to improved patient prognosis [13].

In this review, we comprehensively summarize the clinical manifestations, pathogenesis, diagnostic advances, and management strategies of ocular complications related to HMs, in order to facilitate early interdisciplinary identification and optimization of patient management.

## 2. Methods

This article was conducted as a narrative review. We searched PubMed/MEDLINE for English-language articles published from January 2015 to February 2026 using combinations of the terms “eye disease,” “ocular manifestation,” “hematologic neoplasm,” “leukemia,” and “lymphoma.” Priority was given to articles addressing clinical manifestations, pathogenesis, diagnosis, treatment strategies, and opportunistic infections. Additional relevant references were identified from the bibliographies of eligible articles when appropriate. Because this was a narrative review, no formal systematic-review protocol was applied.

## 3. Classification of Ocular Manifestations in Hematologic Malignancies

Ocular manifestations associated with hematologic malignancies are clinically heterogeneous and arise from multiple pathogenic mechanisms. These manifestations may result from direct infiltration of ocular tissues by malignant hematologic cells or from secondary systemic abnormalities such as anemia, thrombocytopenia, and hyperviscosity. In addition, treatment-related toxicities and opportunistic infections in immunocompromised patients may also contribute to ocular involvement [13]. For clarity, ocular complications can be broadly divided into three major groups: disease-related ocular manifestations, treatment-related ocular manifestations, and secondary infectious ocular manifestations [14]. In the following sections, these categories will be discussed in detail, with particular emphasis on their pathogenesis, clinical presentation, and diagnostic considerations.

### 3.1. Disease-Related Ocular Manifestations

Disease-related ocular manifestations primarily result from the underlying pathophysiology of hematologic malignancies. Malignant hematologic cells can directly infiltrate ocular structures and the systemic abnormalities may affect ocular circulation and retinal integrity [15].

Leukemia and lymphoma represent the major categories discussed in this review. Despite differences in classification, these conditions may share similar ocular manifestations. In leukemia, different subtypes often cause similar manifestations. Therefore, ocular involvement can be categorized according to clinical presentation into retinal manifestations, uveal/choroidal manifestations, orbital and ocular adnexal involvement, anterior segment manifestations, and neuro-ophthalmic manifestations. Lymphoma, in contrast, is commonly classified by site of involvement into ocular adnexal lymphoma and intraocular lymphoma (Table 1).

#### 3.1.1. Leukemia-Associated Ocular Manifestations

Leukemia frequently causes ocular involvement and may affect nearly all ocular structures. Ocular manifestations arise through two principal mechanisms: direct leukemic infiltration of ocular tissues and secondary changes caused by hematologic abnormalities, such as anemia, thrombocytopenia, and hyperviscosity [13]. Structures commonly involved include the retina, uvea, orbit, adnexa, and optic nerve. Clinically, ocular involvement may represent the initial presentation of leukemia or indicate disease recurrence.

- Retinal Manifestations

The retina is the most commonly affected ocular structure in patients with leukemia, and retinal manifestations represent the predominant form of ocular involvement [15]. Direct retinal infiltration may occur when malignant leukemic cells invade retinal tissues. It has been reported in CML and may lead to visual impairment [32,33,34,35]. In some cases, leukemic infiltration has also been associated with retinal detachment [36]. In addition, retinal ischemic changes may occur, likely related to capillary nonperfusion [37]. Although direct retinal infiltration is less common than secondary retinal manifestations, it may indicate systemic disease and requires prompt clinical attention.

**Figure 1:**
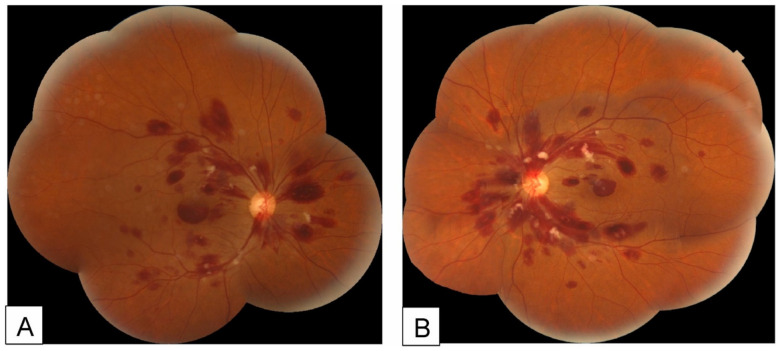
Fundus photographs of the right (**A**) and left (**B**) eyes of a patient with acute leukemia, showing intraretinal and subhyaloid hemorrhages with white-centered retinal hemorrhages (Roth spots). Reproduced from Ben Abdesslem et al. [45] under the Creative Commons Attribution-NonCommercial-NoDerivatives 4.0 International License (CC BY-NC-ND 4.0).

**Figure 2:**
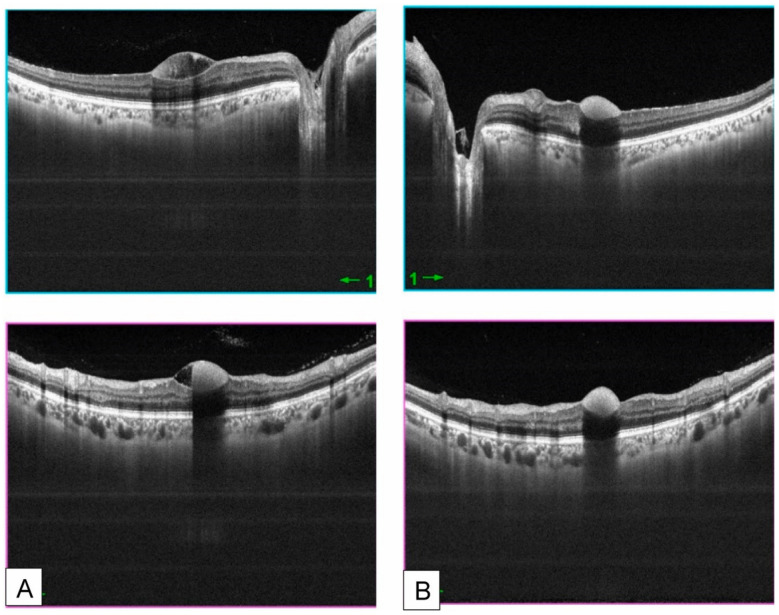
Swept-source optical coherence tomography images of the right (**A**) and left (**B**) eyes of a patient with acute leukemia, demonstrating intraretinal and subhyaloid hemorrhages. Reproduced from Ben Abdesslem et al. [45] under the Creative Commons Attribution-NonCommercial-NoDerivatives 4.0 International License (CC BY-NC-ND 4.0).

Secondary retinal manifestations, collectively termed leukemic retinopathy, are more frequently observed [13]. A study of 102 patients demonstrated that retinal hemorrhage was significantly associated with low hemoglobin levels, elevated total leukocyte count (TLC), and reduced platelet counts, suggesting impaired retinal microcirculation as the underlying mechanism [38]. Clinical manifestations include retinal hemorrhage (including cotton-wool spots and Roth spots) and vascular occlusion. Retinal hemorrhage can be found in almost all types of leukemia [39,40,41,42]. Cotton-wool spots represent focal infarctions of the retinal nerve fiber layer caused by microvascular ischemia [43]. Roth spots, or white-centered retinal hemorrhages, are characterized by round, oval, or flame-shaped hemorrhages with a pale center [44]. The central white area is thought to consist of fibrin–platelet aggregates with entrapped cellular elements following capillary rupture (Figure 1 and Figure 2) [45]. These findings are frequently observed in leukemia [46,47]. In addition, retinal artery and vein occlusions have been reported, possibly related to hyperviscosity [48,49,50].

Leukemic retinal involvement may occasionally mimic necrotizing retinitis, posing a diagnostic challenge [51,52]. This indicates that a thorough differential diagnosis is required to accurately identify potential leukemic involvement. Importantly, retinal manifestations may be the initial symptom of the disease. Therefore, performing fundus examinations in patients with leukemia enables systematic assessment of their clinical condition.

- Uveal and Choroidal Involvement

**Figure 3:**
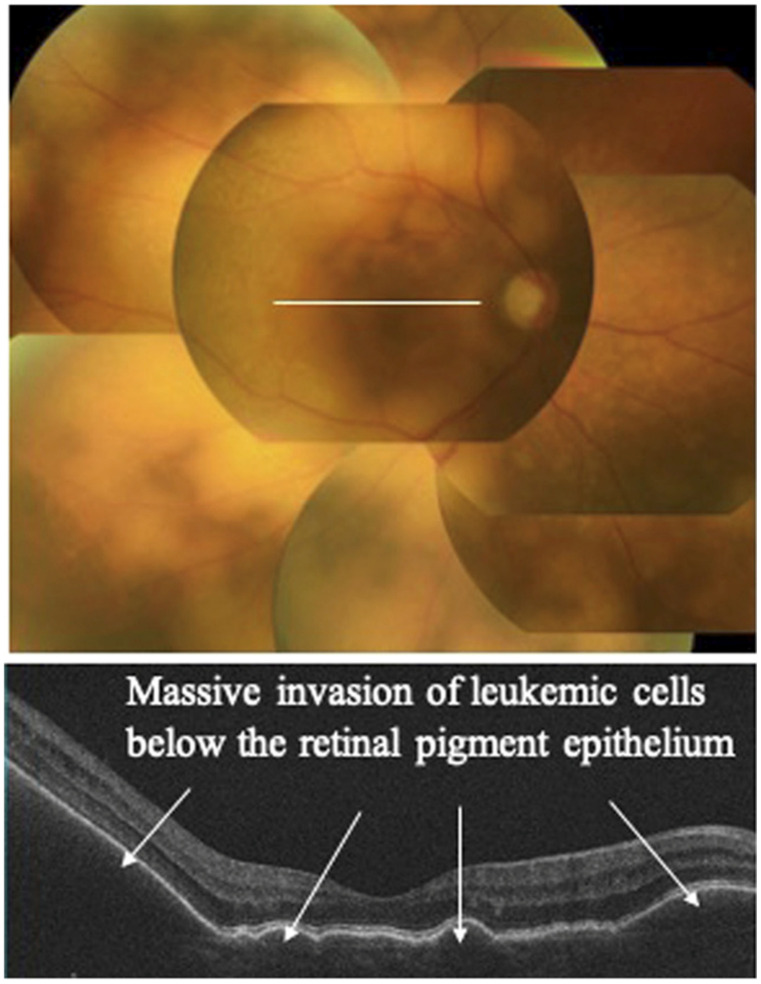
Representative ocular findings of intraocular leukemic infiltration in HTLV-1-associated adult T-cell leukemia/lymphoma. (**upper**) Color fundus photograph showing yellowish-white infiltrative foci associated with retinal protrusions. (**lower**) Optical coherence tomography demonstrating massive solid infiltrative foci beneath the retinal pigment epithelium, consistent with chorioretinal involvement. Reproduced from Kamoi and Ohno-Matsui [61] under the Creative Commons Attribution License (CC BY).

The choroid is particularly susceptible to leukemic infiltration because of its rich vascular supply. Choroidal infiltration may disrupt the function of the retinal pigment epithelium (RPE), leading to serous retinal detachment and subsequent visual impairment [16,17,53]. Among the hematologic malignancies that can involve the uveal tract, adult T-cell leukemia (ATL) represents a rare but clinically important entity because of its association with Human T-cell Leukemia Virus type 1 (HTLV-1) infection [54,55,56,57,58]. HTLV-1 infection is associated with both uveitis and ATL. However, ocular involvement in ATL is uncommon, and HTLV-1–associated uveitis rarely progresses to ATL. In a study of 175 patients with ATL, only three cases of uveitis were reported (Figure 3) [8,59,60,61]. Similarly, among 132 patients with HTLV-1–associated uveitis, only three developed ATL during follow-up [59]. One proposed mechanism is that ATL cells disrupt the blood–ocular barrier, thereby inducing intraocular inflammation [62]. However, given the limited number of reported cases, further studies are required to clarify this association.

- Orbital and Adnexal Involvement

Leukemic involvement of the orbit and ocular adnexa represents a form of direct extramedullary infiltration and is most commonly observed in acute leukemia. Orbital involvement is particularly frequent in pediatric patients and may occasionally serve as the initial manifestation of the disease [18,20,63]. Orbital masses caused by leukemic infiltration can produce a range of clinical features, including proptosis, eyelid swelling, ptosis, and diplopia due to compression of adjacent structures. Chloroma, also known as myeloid sarcoma, is a characteristic extramedullary manifestation of AML. It most commonly involves the orbit and may be asymptomatic in up to 50% of cases. When symptomatic, clinical features depend on the size and location of the lesion and may include pain, mass effect, and organ dysfunction [19]. In addition to orbital lesions, leukemic infiltration may also affect ocular adnexal structures such as the eyelids and lacrimal gland, contributing to periocular swelling or mass-like lesions [64].

- Anterior Segment Manifestations

**Figure 4:**
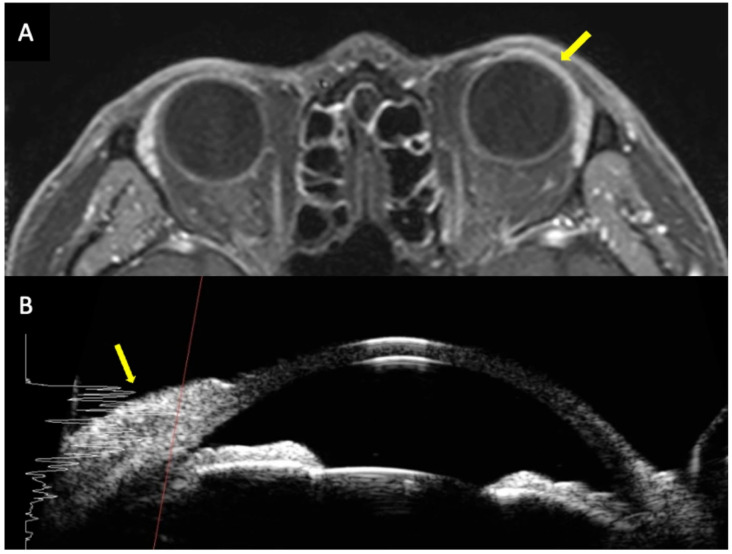
A patient with acute myeloid leukemia presenting with conjunctival lesions. Orbital MRI (**A**) and ultrasound biomicroscopy (**B**) show linear thickening and hyperreflectivity (yellow arrows). Reproduced from Park et al. [66] under the Creative Commons Attribution 4.0 International License (CC BY 4.0).

Anterior segment manifestations in leukemia are relatively uncommon. However, several manifestations have been reported. Direct infiltration may affect structures such as the conjunctiva, iris, and sclera. Scleral nodules have been described as a rare manifestation in patients with CLL [65]. Conjunctival infiltration has also been reported in certain leukemia subtypes and may contribute to anterior segment abnormalities (Figure 4) [22,66,67]. Secondary complications may also occur. In a cohort study of patients with ALL, the incidence of ocular hypertension was reported to be as high as 61.1% [21]. Elevated intraocular pressure may be related to leukemic infiltration of ocular tissues, which can impair aqueous humor outflow [68]. In addition, rare anterior segment involvements have been described. For example, iris pigment epithelial cysts have occasionally been reported in patients with ALL [69].

- Neuro-ophthalmic Manifestations

Neuro-ophthalmic manifestations in leukemia may arise from direct leukemic infiltration of the optic nerve or from secondary effects associated with central nervous system (CNS) involvement. Direct infiltration of the optic nerve by leukemic cells may lead to optic disc edema and progressive visual impairment. In some cases, the recurrence of ALL may present with optic nerve infiltration as the initial symptom [24,70]. Prompt treatment may result in significant visual recovery [71]. CNS involvement is a major contributor to morbidity and mortality in pediatric acute leukemia, particularly in ALL [23]. Increased intracranial pressure due to CNS infiltration may lead to papilledema, which presents as bilateral optic disc swelling and visual disturbances [72,73].

Reported prevalence of ocular manifestations varies substantially across leukemia subtypes. A retrospective study reported that ocular manifestations were more frequent in acute leukemia (51.9%) than chronic leukemia (25%), and ocular manifestations were significantly more frequent in myeloid leukemia subtypes (52.9%) than lymphoid leukemia subtypes (28.6%) (Table 2) [74].

#### 3.1.2. Lymphoma-Associated Ocular Manifestations

Lymphoma is one of the most common hematologic malignancies associated with ocular manifestations [26]. Ocular manifestations are mainly associated with NHL and may occur as either primary or secondary [80]. Ocular involvement can affect multiple structures, including the vitreous, retina, uvea, orbit, and ocular adnexa. Based on anatomical location, ocular lymphoma is broadly classified into intraocular lymphoma and ocular adnexal lymphoma. In this section, lymphoma-associated ocular manifestations are summarized according to the involved anatomical structures.

- Vitreoretinal Lymphoma

Vitreoretinal lymphoma (VRL) is a subtype of primary central nervous system lymphoma (PCNSL), a form of lymphoma that originates within the central nervous system. Most cases of VRL are diffuse large B-cell lymphoma (DLBCL), whereas T-cell VRL is rare. VRL may present as primary intraocular lymphoma or as secondary involvement associated with CNS disease. Despite differences in origin, these forms share similar pathological features, characterized by malignant lymphocytic proliferation with infiltration of the vitreous and retina [25]. Experimental studies suggest that chemokine receptors, such as CXCR4 and CXCR5, and adhesion molecules including CD44 may contribute to lymphoma cell migration into ocular tissues [81]. Clinically, VRL frequently presents with bilateral involvement. The vitreous is most commonly affected, whereas isolated retinal involvement is uncommon [82]. Characteristic findings include dense vitreous cellular infiltration and multiple cream-colored subretinal lesions on fundus examination [25]. In the early stages, VRL often masquerades as uveitis, particularly anterior or intermediate uveitis, which may result in delayed diagnosis [83]. Cytologic or histopathologic identification of malignant B cells, confirmed by immunophenotyping, remains the gold standard for diagnosis. In addition, molecular testing has demonstrated value in cases with limited or poor-quality samples [27].

- Ocular Adnexal Lymphoma

Ocular adnexal lymphoma mainly occurs in the conjunctiva, eyelid, orbit, and lacrimal gland. Lymphoma is the most common malignancy of the ocular adnexa, and its main manifestation is orbital lymphoma [29]. Similar to VRL, the majority of ocular adnexal lymphomas originate from B cells, but there are also a few cases reported involving NK cells or T cells [84,85]. Depending on the involved structures, there are certain differences in the clinical manifestations. Conjunctival lymphoma may present with pink conjunctival masses or conjunctival infiltration, often described as a “salmon patch” [22,86]. B-cell lymphoma of the eyelid typically presents as eyelid swelling or tumor. The presence of proptosis may indicate orbital involvement. Meanwhile T-cell lymphoma mainly manifests as ulceration and erythema [87]. The main manifestations of orbital lymphoma include palpable masses, accompanied by vision loss, proptosis, motility disturbances and diplopia [88]. The manifestations of lacrimal gland lymphoma are similar to those of orbital lymphoma, which may be accompanied by pain and involvement of other structures [89].

For ocular adnexal lymphoma, imaging findings alone are insufficient for definitive diagnosis; however, MRI with diffusion-weighted imaging may provide useful supportive information, because lower ADC values tend to favor lymphoma over benign orbital lymphoproliferative disorders. Histopathologic biopsy remains essential for definitive diagnosis and subtype classification [29,90].

#### 3.1.3. Other Hematologic Malignancies with Ocular Involvement

Although this review mainly focuses on leukemia and lymphoma, other hematologic malignancies may also be associated with clinically relevant ocular manifestations.

Myelodysplastic syndromes (MDS) are a very heterogeneous group of myeloid disorders characterized by peripheral blood cytopenias and increased risk of transformation to AML [91]. MDS is regarded as a premalignant myeloid disorder, and some ocular manifestations overlap with those observed in leukemia. The main ocular manifestations of MDS include retinal hemorrhages [92]. However, several rare case reports have described that sudden increases in intraocular pressure, optic neuritis, and choroidal infiltration may also occur [93,94,95].

Multiple myeloma (MM) is a malignant plasma cell neoplasm. It is characterized by clonal proliferation of plasma cells in the bone marrow [96]. Because of its rich vascular supply and abundant marrow space, MM typically involves the posterior extraconal space of the orbit, with a predilection for the superotemporal quadrant [97]. Multiple myeloma can also cause hyperviscosity, which in turn leads to retinal vein occlusion [98]. MM may also involve the lacrimal gland, cornea, and other periocular tissues [99,100]. MM may rarely present with iris plasmacytoma, but it is extremely rare [101].

### 3.2. Treatment-Related Ocular Manifestations

Advances in the treatment of hematologic malignancies, including chemotherapy, targeted therapy, immunotherapy, and hematopoietic stem cell transplantation, have significantly improved patient survival. However, these therapeutic approaches may also lead to a range of ocular complications. These treatment-related ocular manifestations may result from direct drug toxicity, immune-mediated reactions, or treatment-induced immunosuppression leading to opportunistic infections.

#### 3.2.1. Chemotherapy-Related Toxicity

With increasing use of chemotherapy, treatment-related ocular manifestations have become increasingly recognized. Although these manifestations are relatively uncommon, they are clinically significant [102]. Ocular toxicity can manifest in the eye and ocular adnexa. Cytarabine has been commonly associated with conjunctivitis and keratitis. Methotrexate may also cause ocular manifestations, including epiphora, blepharitis, conjunctivitis, and cataracts [102]. Neuro-ophthalmic manifestations, although less common, represent potentially vision-threatening adverse events. Methotrexate may also cause optic nerve toxicity, leading to demyelinating lesions, which might be related to its interference with folate metabolism [103]. Vincristine’s toxicity is neurologic, which may cause ptosis in patients [104]. The use of neurotrophic/neuroprotective agents such as pyridoxine and pyridostigmine may accelerate recovery [105]. Overall, oncologists should always consider that ocular issues may be related to the chemotherapy treatment [106].

#### 3.2.2. Targeted Therapy-Related Toxicity

**Figure 5:**
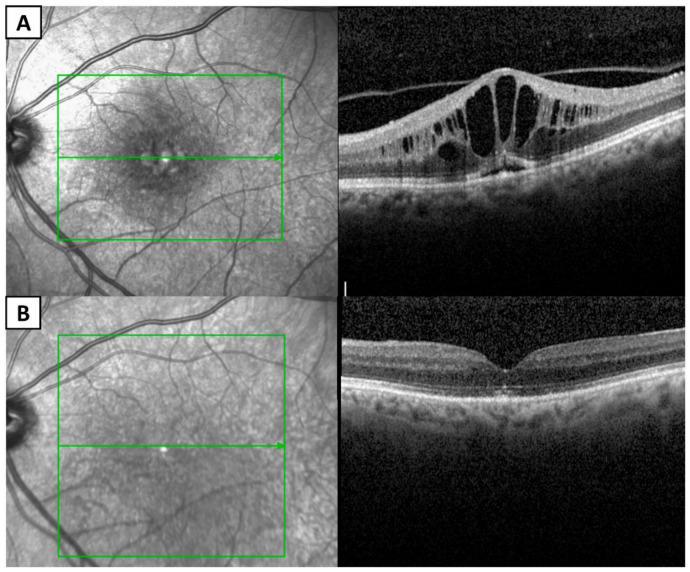
Optical coherence tomography images showing cystoid macular edema in the left eye during ibrutinib treatment (**A**) and resolution of the cystoid macular edema 6 months after cessation of ibrutinib (**B**). Subretinal hyperreflective material is visible in both images. The green square and arrows delineate the location and extent of the lesion. Reproduced from Ben-Avi et al. [111] under the Creative Commons Attribution-NonCommercial-NoDerivatives 4.0 International License (CC BY-NC-ND 4.0).

Targeted therapies have become an important component of the management of hematologic malignancies. Although these agents are generally better tolerated than conventional chemotherapy, they may still cause a variety of ocular adverse effects [107]. These complications may result from off-target effects, immune modulation, or drug accumulation in ocular tissues. Imatinib, a tyrosine kinase inhibitor (TKI), has been widely used in hematologic malignancies [7]. It has significantly improved outcomes in CML. The most common ocular adverse effect of imatinib is periorbital edema. In addition, epiphora, optic neuritis, and cystoid macular edema may also occur. These ocular manifestations may result from increased capillary permeability and fluid extravasation induced by imatinib [108]. Recent case reports have described imatinib-induced myasthenia gravis presenting with ptosis of the eyelid, which may be related to immune dysfunction [109]. Ibrutinib, a Bruton tyrosine kinase (BTK) inhibitor, is widely used in CLL. Ibrutinib has been reported to cause ocular complications, including uveitis and cystoid macular edema in some patients (Figure 5) [110,111,112]. Antibody–drug conjugates are a newer therapeutic class to enhance the efficacy of chemotherapy drugs and reduce their toxicity. However, they can still cause ocular toxicity [113]. Therefore, clinicians should remain vigilant for ocular adverse effects in patients receiving targeted therapies.

#### 3.2.3. Immunotherapy-Related Toxicity

Immunotherapy, including immune checkpoint inhibitors (ICIs) and chimeric antigen receptor T-cell (CAR-T) therapy, has significantly improved outcomes in hematologic malignancies through immune activation [114]. However, immunotherapy may lead to off-target immune-mediated injury in other organs or tissues, resulting in immune-related adverse events (irAEs) [115,116].

The ocular manifestations associated with ICIs are diverse and can be broadly classified into two categories. The first category includes neuro-ophthalmic and orbital disorders, such as immune-related optic neuritis, inflammatory optic disc edema, and orbital inflammation. The second category includes uveitis and ocular surface disorders, such as immune-related uveitis, Vogt–Koyanagi–Harada-like syndrome, and dry eye disease [116].

In contrast to ICIs, ocular manifestations following CAR-T therapy appear to be relatively uncommon; however, recent pharmacovigilance data suggest that such complications may be underrecognized. In a recent FAERS-based study, 53 ocular adverse events were identified after CAR-T therapy, including visual disturbance, vitreous opacity, diplopia, and xerophthalmia [12]. The most commonly reported manifestations are visual changes, including vitreous opacities, visual disturbances, diplopia, and visual discomfort. Inflammation and dry eye syndrome may also occur [117]. CAR-T therapy may not only directly affect the ocular structures, but its neurotoxicity may also impact the optic nerve, leading to ocular symptoms [118].

Overall, immunotherapy-related ocular toxicities likely reflect dysregulated immune activation affecting multiple ocular tissues. Therefore, prompt recognition of ocular adverse events during immunotherapy is essential to prevent treatment-related visual morbidity.

#### 3.2.4. Hematopoietic Stem Cell Transplantation (GVHD)

Hematopoietic stem cell transplantation (HSCT) is an important therapeutic strategy for various hematologic malignancies [119]. However, transplantation-related complications may affect multiple organs, among which graft-versus-host disease (GVHD) is one of the most important immune-mediated complications. Ocular GVHD (oGVHD) results from donor T-cell-mediated immune responses against host ocular tissues. The thymus and lymphoid tissues may fail to adequately eliminate donor self-reactive T cells (CD4^+^ and CD8^+^), allowing persistence of autoreactive donor T cells. These T-cell-mediated immune responses are directed against host antigens against major (MHC) and minor (miHAG) histocompatibility antigens. After transplantation, the differences in host and donor antigen expression activate the donor T cells, triggering inflammatory cascades and cytokine release. The host antigen-presenting cells (APC) can also drive activation of donor T cells, and donor B cells may also contribute to sustained immune activation. oGVHD can be classified into acute and chronic forms. Acute oGVHD mainly presents with conjunctival symptoms. Chronic oGVHD presents with broader manifestations, which can involve the conjunctiva, cornea, eyelids, and lacrimal glands [120].

In a study of 620 patients undergoing allogeneic HSCT, approximately 13% developed oGVHD, making it one of the most common complications [121]. Therefore, the diagnosis and treatment of ocular complications are of great importance. According to the International Chronic Ocular Graft-vs-Host-Disease (GVHD) Consensus Group, diagnosis of ocular GVHD is based on 4 parameters: (1) Ocular Surface Disease Index (OSDI) score; (2) Schirmer test; (3) corneal fluorescein staining; (4) conjunctival injection. Each variable was scored 0–2 or 0–3, with a maximum composite score of 11. The diagnosis is categorized as no, probable, or definite ocular GVHD based on the total score and the presence of systemic GVHD [122].

To detect oGVHD early, regular and comprehensive ocular examinations are required [123]. For patients with elevated total bilirubin and γ-glutamyl transferase (GGT), more detailed examinations are necessary, as these patients are at higher risk of developing oGVHD [124].

### 3.3. Secondary Infectious Complications

Patients with hematologic malignancies are highly susceptible to opportunistic infections due to disease-related immune dysfunction as well as treatment-induced immunosuppression. These infections may involve various ocular structures and can lead to severe visual impairment if not recognized early [125].

Viral infections represent one of the most common infectious complications. Cytomegalovirus (CMV) retinitis has been frequently reported in immunocompromised patients and is characterized by necrotizing retinitis with retinal hemorrhages and vascular involvement [126,127,128,129,130]. The decrease in CD4^+^ T cell levels may suggest its occurrence, which might be helpful for its diagnosis [131]. Fungal infections are another important cause of ocular complications in patients with hematologic malignancies. The main manifestation is fungal endophthalmitis. It can be observed that both molds and yeasts can act as pathogenic agents [132,133,134,135,136,137]. Although less common, bacterial infections may also occur in severely immunocompromised patients, occasionally leading to uveitis [138,139]. Secondary infections often involve the retina. If not treated promptly, they may lead to blindness. Therefore, early recognition and prompt antimicrobial therapy are essential to prevent irreversible ocular damage.

## 4. Clinical Implications and Diagnostic Considerations

Ocular manifestations in hematologic malignancies represent a complex clinical spectrum that extends beyond isolated ophthalmic complications. As summarized in the preceding sections, ocular manifestations may arise from direct infiltration, treatment-related toxicity, or secondary infections in the setting of immunosuppression. Importantly, these ocular manifestations are not just local events but may reflect systemic disease activity, treatment response, or disease recurrences. The heterogeneous and often nonspecific nature of ocular manifestations poses significant diagnostic challenges and increases the risk of delayed diagnosis. Therefore, understanding the clinical implications of ocular manifestations is essential for timely diagnosis, appropriate management, and improved visual and systemic prognosis.

### 4.1. Diagnostic Considerations

Ocular involvement in patients with hematologic malignancies may present with a wide range of symptoms, including blurred vision, ocular pain, and visual field defects. In some cases, ocular manifestations may represent the initial presentation of the underlying malignancy or indicate disease progression or relapse [140]. Therefore, early recognition and comprehensive ophthalmic evaluation are essential for timely diagnosis and appropriate management.

A thorough ophthalmic examination is the cornerstone of diagnosis. Slit-lamp examination allows detailed assessment of the anterior segment and may reveal inflammatory changes or tumor infiltration involving the cornea and iris [141]. Fundus examination remains particularly important for detecting posterior segment abnormalities such as Roth spots, a type of retinal hemorrhage.

In addition to clinical examination, ophthalmic imaging techniques play an important role in the evaluation of ocular manifestations. Optical coherence tomography (OCT) provides high-resolution cross-sectional imaging of retinal structures and is useful for detecting retinal infiltration, macular edema, or subretinal lesions [32].

MRI can be used to assess optic nerve infiltration by hematologic malignancies and involvement of the central nervous system [142]. For ocular adnexal lymphoma, MRI with diffusion-weighted imaging may facilitate differentiation from benign orbital lymphoproliferative disorders. Orbital lymphoma tends to show lower baseline apparent diffusion coefficient (ADC) values compared with benign lesions. Therefore, ADC measurements may be useful for diagnosis and prediction of therapeutic response [30]. Some MRI features can also be helpful in differentiating orbital lymphoma from benign orbital lymphoproliferative disorders. Ill-defined tumor margins were significantly associated with orbital lymphoma, whereas “flow void sign” and radiologic evidence of sinusitis (*p* = 0.0002) were associated with benign orbital lymphoproliferative disorders [31].

In selected cases, particularly when intraocular lymphoma or other hematologic malignancy-related masquerade syndromes are suspected, diagnostic procedures such as vitreous biopsy, cytological analysis, flow cytometry, and ocular fluid PCR may be required to confirm the diagnosis [143,144]. Overall, accurate diagnosis relies on a combination of clinical findings, imaging modalities, and laboratory investigations. PCR-based analysis of ocular fluids may be particularly useful when infectious uveitis or masquerade syndromes are part of the differential diagnosis [144]. When endogenous fungal endophthalmitis is suspected, molecular testing of ocular fluids may provide useful microbiologic support [145].

### 4.2. Diagnostic Challenges and Risk of Misdiagnosis

Misdiagnosis of ocular manifestations associated with hematologic malignancies may lead to serious clinical consequences. These conditions, particularly lymphoma, often masquerade as uveitis [146]. In such cases, corticosteroid therapy administered for presumed uveitis may temporarily alleviate inflammation while masking the underlying malignancy, thereby delaying accurate diagnosis. For example, one case report described a patient initially diagnosed with uveitis and treated accordingly; however, the correct diagnosis of vitreoretinal lymphoma was established only after 7 months, when symptoms progressively worsened [147]. Similarly, there have been reports of cases where leukemia relapse presents as masquerade uveitis [148]. This suggests that if uveitis persists despite treatment and fails to improve, it should be suspected as masquerade uveitis.

### 4.3. Importance of Multidisciplinary Collaboration and Follow-Up

Hematologic malignancies can present various ocular manifestations. Effective treatment requires close collaboration between hematologists and ophthalmologists. Ocular manifestations can serve as indicators of systemic recurrence or the toxicity of the treatment, and timely adjustment of the treatment strategy is necessary. Multidisciplinary collaboration is therefore essential for improving patient outcomes.

Regular follow-up and ophthalmic screening are critical in patients with hematologic malignancies. These measures facilitate early detection of disease recurrence as well as treatment-related adverse effects. For example, during routine follow-up after hematopoietic stem cell transplantation, ocular graft-versus-host disease can be identified and managed at an early stage [124]. These observations highlight that the management of hematologic malignancies is a long-term process. Early recognition of ocular manifestations may provide important clinical insights and guide overall patient management.

## 5. Treatment Strategies

Management of ocular manifestations in hematologic malignancies primarily focuses on controlling the underlying systemic disease while preserving visual function. In some cases, effective systemic therapy can lead to improvement of ocular manifestations [149]. However, local ophthalmic treatment is often required when ocular manifestations persist or when vision-threatening complications arise. In this section, we summarize systemic therapy, local ocular treatment, and the management of infectious complications.

### 5.1. Systemic Treatment

Systemic therapy targeting the underlying hematologic malignancy is often essential for controlling ocular manifestations. Chemotherapy, targeted therapy, and immunotherapy have significantly improved patient outcomes and may lead to resolution of ocular manifestations. For example, after systemic chemotherapy (hydroxyurea, allopurinol) combined with targeted therapy (imatinib), ocular manifestations in patients with CML may show significant improvement [150]. Systemic treatment of VRL may reduce the risk of subsequent central nervous system lymphoma [143].

### 5.2. Local Ocular Treatment

Local ocular therapy is indicated in selected cases to directly control intraocular disease. Combined systemic and local therapy has been associated with improved outcomes in primary vitreoretinal lymphoma [28]. A commonly used regimen consists of repeated intravitreal methotrexate 400 μg/0.1 mL, twice weekly in the first month, once weekly in the following two months and once in a month thereafter. This treatment regimen can alleviate ocular inflammatory responses and reduce leukemic infiltration [151]. Twice-weekly intravitreal methotrexate for a total of eight injections has been reported to be the most effective and least harmful treatment with a very low local recurrence rate (2%) and limited and manageable side effects [143]. Evidence from two studies suggests that combined intravitreal injection and systemic treatment can significantly extend disease-free survival [152,153]. However, another cohort study found that combined intravitreal and systemic therapy did not affect the risk of central nervous system or systemic lymphoma progression, ocular disease relapse and overall survival [154]. Therefore, further research is still needed to understand the effects of combined treatment.

### 5.3. Management of Infectious Complications

Patients with hematologic malignancies are at increased risk of ocular infections, which require prompt and targeted treatment. Management depends on the causative pathogen. Systemic antiviral therapy is commonly used for viral infections such as cytomegalovirus retinitis [129,130]. Intravitreal antiviral injections may provide additional therapeutic benefit in severe cases [128]. For fungal infections, both systemic and intraocular antifungal therapies are indicated, and vitrectomy may be considered when necessary [132].

## 6. Conclusions

In summary, ocular manifestations of hematologic malignancies have substantial diagnostic and management implications. They encompass a broad spectrum of findings, including retinal hemorrhages, vision loss, vitreoretinal infiltration, and orbital masses. Importantly, these manifestations may represent the initial presentation of an undiagnosed malignancy, a clue to systemic relapse, or a complication of treatment.

From a clinical perspective, several ocular findings warrant particular attention. Findings such as retinal hemorrhages, Roth spots, unexplained optic disc edema, or uveitis refractory to standard therapy should prompt ophthalmologists to consider an underlying hematologic malignancy or relapse. If a patient with leukemia develops recurrent retinal hemorrhage or worsening vision despite apparent hematologic improvement, relapse should be considered. Early ophthalmic and hematologic evaluation, together with close collaboration between ophthalmologists and hematologists, are essential to preserve vision and improve prognosis.

Most of the ocular manifestations improve as the systemic symptoms are controlled. However, some patients may benefit from local ocular therapy, including intravitreal chemotherapy or radiotherapy. During treatment, clinicians must remain alert to treatment-related ocular toxicities associated with targeted therapies, immunotherapies, hematopoietic stem cell transplantation, and immunosuppression.

Ocular manifestations are not only indicators of recurrence, but also toxic effects of therapy. Regular ophthalmic evaluation should be considered in patients with hematologic malignancies, particularly those with visual symptoms, high-risk systemic disease, hematopoietic stem cell transplantation, or therapies associated with ocular toxicity.

However, the current evidence base remains limited. Most available data derive from retrospective studies, case series, or geographically concentrated cohorts, with few prospective comparative trials. Standardized screening protocols, validated risk stratification tools, and consensus treatment algorithms are still lacking.

Future research should prioritize multicenter prospective studies for standardized evaluations of ocular toxicity, and clarify predictors of relapse-related ocular manifestations. With the advent of new therapies, the survival of patients with hematologic malignancies is gradually improving. Preserving long-term visual function and quality of life will become an increasingly important component of care of patients with hematologic malignancies.

## Figures and Tables

**Table 1:** Disease-related ocular manifestations of leukemia and lymphoma.

Disease	Ocular Structure Involved	Main Clinical Manifestations	Pathogenesis	Prevalence	Diagnosis	Management	References
Leukemia	Retina	Retinal hemorrhages, Roth spots, cotton-wool spots, venous tortuosity, vascular occlusion	Secondary Hematologic Abnormalities	30–50%	Fundus exam, OCT	Systemic treatment of the underlying malignancy	[13,14,15]
	Uvea and Choroid	Serous retinal detachment, blurred vision, uveitis-like inflammation	Leukemic infiltration with RPE dysfunction, blood stagnation or mechanical compression disrupts the intercellular tight junctions	High prevalence in autopsy	Fundus exam, OCT	Systemic and intrathecal chemotherapy, local irradiation	[14,16,17]
	Orbit and Adnexa	Proptosis, eyelid swelling, ptosis, diplopia, orbital mass, chloroma	Extramedullary leukemic infiltration	Less common	MRI, CT, B-scan, biopsy	Timely initiation of chemotherapy	[18,19,20]
	Anterior Segment	Ocular hypertension, conjunctival infiltration, iris lesions	Leukemic infiltration impairs aqueous humor outflow	Rare	IOP measurement, biopsy	IOP lowering and systemic treatment	[21,22]
	Optic Nerve	Papilledema, optic disc edema, optic nerve infiltration	Central nervous system infiltration, increased blood viscosity, a compressive phenomenon	About 17.4%	Fundus exam, MRI	Intrathecal chemotherapy, cranial irradiation	[23,24]
Lymphoma	Vitreous Body and Retina (Primary Vitreoretinal Lymphoma)	Vitritis, masquerade uveitis	An immune-privileged ocular microenvironment, an IL-10-dominant cytokine profile, and lymphoma-cell migration mediated by adhesion molecules and chemokine receptors may contribute to disease development.	Rare	MRI, cytokine analysis, cytology, flow cytometry, molecular analysis	Local treatment, systemic chemotherapy, Radiotherapy, Intravitreal methotrexate	[25,26,27]
	Vitreous Body and Retina (Secondary intraocular lymphoma)	Blurred vision and floaters, Vitreous hemorrhages, neuro-ophthalmic manifestations	Hematogenous spread of systemic lymphoma cells into ocular tissues	Rare in VRL	MRI, biopsy	Systemic chemotherapy with or without local radiotherapy/intravitreal therapy, depending on disease extent	[26,28]
	Orbit and Adnexa	Proptosis, diplopia, palpable mass, motility disturbance, vision loss	Acquired genetic alterations and, in some cases, chronic antigenic stimulation or immunologic dysregulation may contribute to disease development.	50–60% of ocular adnexal lymphomas	DWI, MRI, biopsy	Antibiotics, Radiotherapy, systemic chemotherapy	[29,30,31]

**Table 2:** Reported prevalence of ocular manifestations across leukemia subtypes.

Subtypes	Prevalence	Most Common Manifestation	Clinical Notes	References
AML	39–67% [74,75,76,77]	Roth spots, cotton-wool spots, vascular tortuosity and dilatation, microaneurysms and neovascularizations	Higher with posterior segment involvement	El Salloukh et al. [10]
ALL	15–43% [74,75,76,77]	Ocular hypertension, retinal hemorrhage, vision loss	Ocular manifestations are often silent	de Queiroz Mendonca et al. [21]
CML	5–10% [78]	Leukemic retinopathy, proliferative retinopathy	Ischemic-like retinopathy may be the initial symptom	Yassin et al. [15]
CLL	Uncommon	CLL infiltration, Richter transformation, infection	Ophthalmic features are rare and often initially misdiagnosed	Delestre et al. [79]

## Data Availability

No new data were created or analyzed in this study. Data sharing is not applicable to this article.
